# Age Correction in Dementia – Matching to a Healthy Brain

**DOI:** 10.1371/journal.pone.0022193

**Published:** 2011-07-29

**Authors:** Juergen Dukart, Matthias L. Schroeter, Karsten Mueller, The Alzheimer's Disease Neuroimaging Initiative

**Affiliations:** 1 Max Planck Institute for Human Cognitive and Brain Sciences, Leipzig, Germany; 2 Day Clinic of Cognitive Neurology, University of Leipzig, Leipzig, Germany; 3 LIFE - Leipzig Research Center for Civilization Diseases, University of Leipzig, Germany; Cuban Neuroscience Center, Cuba

## Abstract

In recent research, many univariate and multivariate approaches have been proposed to improve automatic classification of various dementia syndromes using imaging data. Some of these methods do not provide the possibility to integrate possible confounding variables like age into the statistical evaluation. A similar problem sometimes exists in clinical studies, as it is not always possible to match different clinical groups to each other in all confounding variables, like for example, early-onset (age<65 years) and late-onset (age≥65) patients with Alzheimer's disease (AD). Here, we propose a simple method to control for possible effects of confounding variables such as age prior to statistical evaluation of magnetic resonance imaging (MRI) data using support vector machine classification (SVM) or voxel-based morphometry (VBM). We compare SVM results for the classification of 80 AD patients and 79 healthy control subjects based on MRI data with and without prior age correction. Additionally, we compare VBM results for the comparison of three different groups of AD patients differing in age with the same group of control subjects obtained without including age as covariate, with age as covariate or with prior age correction using the proposed method. SVM classification using the proposed method resulted in higher between-group classification accuracy compared to uncorrected data. Further, applying the proposed age correction substantially improved univariate detection of disease-related grey matter atrophy using VBM in AD patients differing in age from control subjects. The results suggest that the approach proposed in this work is generally suited to control for confounding variables such as age in SVM or VBM analyses. Accordingly, the approach might improve and extend the application of these methods in clinical neurosciences.

## Introduction

In recent research, age-related changes have frequently been reported in different imaging modalities investigating healthy subjects and patients in advanced age [1]–[4]. When comparing groups of younger and older subjects, most studies have reported age-related decreases in grey matter (GM) densities in specific brain structures [1],[4],[5] measured by magnetic resonance imaging (MRI). Age-related changes have also been reported for functional measurements like glucose utilization [6] measured by [18F]fluorodeoxyglucose positron emission tomography. The use of different univariate and multivariate statistical approaches for the comparison of different groups of dementia patients with healthy control subjects has led to the necessity to control for age-related changes, as these might cover or lead to an overestimation of group-specific differences.

Usually, when comparing imaging data of different groups of subjects, these are matched for such confounding effects as age and sex, and the confounding variable is usually integrated as a covariate in the statistical model [7]. However, when using multivariate approaches, for example for automatic detection or differentiation of types of dementia, it is not always possible to control for age-related changes or any other confounding variables. Multivariate classification algorithms do not usually provide the possibility to integrate covariates into between-group classification.

As has been shown by Franke et al. [8], age estimation in patients with mild Alzheimer's disease (AD) based on T1-scans results in an age gap of +10 years for these subjects compared to healthy control subjects. Age of AD patients is overestimated using T1-scans because age-related changes are equally directed with changes associated with AD. As a result of the equal direction of these changes, the classification algorithm might also lead to a misclassification of younger AD patients and older control subjects. Therefore, to avoid these misclassifications, groups used for the training of the multivariate pattern classifier should ideally be matched to each single subject who has to be classified in clinical setting. This issue is highly important for clinical practice as the use of all univariate and multivariate approaches has mainly the aim of enabling accurate and early detection and differentiation of various dementia syndromes in single subjects.

A similar problem arises when individual dementia patients or two groups of dementia patients differing in age (e.g. early- and late-onset AD) are compared with a specific group of control subjects or to each other using univariate approaches. This is because it is rarely possible to find sufficiently large groups of control subjects, which match each individual patient in age and other confounding variables. Otherwise, when a patient or a group of patients differs from the control group in specific confounding variables, including these into statistical evaluation might cover disease-related changes. This issue might therefore be relevant for group comparisons, as for example when comparing early- and late-onset AD patients with healthy control subjects or to each other it might be interesting to look for differential atrophy patterns in these two groups. By definition, these two groups differ in their mean age (early-onset AD: age<65years, late-onset AD: age ≥65 years). In order to exclude confounding effects of age, two different groups of healthy control subjects are usually used to evaluate atrophy patterns in both AD patients groups [9]. However, this procedure restricts the quantitative and qualitative comparison of atrophy patterns in both groups of AD patients as there might be substantial differences between groups of control subjects used for both comparisons. Therefore, it is highly important to have methodical approaches enabling control for such confounding effects.

In this work, we propose a linear detrending method in terms of the general linear model (GLM) based only on control subjects to control for the effects of age in single subjects and in groups of subjects prior to statistical evaluation using support vector machine classification (SVM) or voxel-based morphometry (VBM). A linear model was chosen based on a study by Good et al. [1]. Thereby, the authors compared linear vs. quadratic models in terms of describing absolute and relative age related GM changes in a healthy cohort consisting of 465 subjects. While the quadratic coefficients failed to reach significance the linear coefficient was highly significant. To evaluate this method, we compare SVM and VBM results for differentiation of AD patients and healthy control subjects with and without linear detrending of grey matter (GM) values for age prior to statistical evaluation. We hypothesize that applying linear age detrending prior to statistical evaluation should increase the diagnostic accuracy for differentiation of dementia patients and control subjects using SVM, and improve univariate detection of GM changes when applying VBM for the evaluation of groups of AD patients differing in age from the control group.

## Methods

### Age correction

To remove age-related effects, it is highly important to differentiate between them and disease-related changes. For that reason, when comparing single AD patients and control subjects using SVM or VBM we propose performing additional GLMs prior to the final statistical evaluation using the same methodical approach as proposed by Friston et al. [7], but only for control subjects. Thereby, GLMs are calculated for all GM voxels 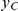 at each coordinate separately. A constant and age are the only columns in the matrix 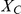 and only the group of healthy control subjects is used to determine the regression coefficients 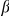, consisting of 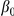 for the constant and 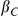, for age-related changes at each voxel coordinate separately. In terms of GLM, the following simple regression model has to be solved for 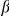 by minimizing the sum of squared residuals , 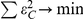:
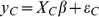
(1)Solving (1) for least squares estimates of 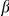 satisfies the following normal equations ([10], p. 91):
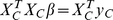
(2)Solving linear equations system (2) for 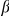 results in:
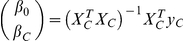



To obtain age-corrected GM values 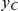, the calculated age regression coefficients 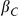 are then applied to corresponding voxels in GM images of both healthy control subjects and AD patients. Thereby, the residual amount explained by each subject's individual age 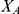 is removed from the observed GM voxel values 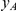 of this subject at each coordinate using the determined coefficient 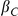, so:
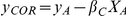



It is very important to use only control subjects to determine the regression coefficient, as it has been reported that early- and late-onset AD patients might show a differential pattern of atrophy in MRI [9],[11,[12]. As a result, removing age-related effects using regression coefficients determined in the AD group might also remove disease-related changes due to their interaction with age. In further statistical analyses, SVM and VBM results obtained using GM images containing initial uncorrected GM values 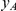 are compared with results using age-corrected values 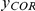. The age correction procedure was implemented in Matlab 7.7 (MathWorks Inc., Sherborn, MA).

### Subjects

To evaluate the effect of age correction, we extracted multicenter MRI data of 80 patients with clinically validated AD and 79 healthy control subjects (Table 1) from the Alzheimer's disease Neuroimaging Initiative (ADNI) database (www.adni-info.org). AD patients were randomly selected from the database. Control subjects were selected to match the AD patients for gender, age and education. The ADNI is a partnership of the National Institute of Aging, the National Institute of Biomedical Imaging and Bioengineering, the Food and Drug Administration, private pharmaceutical companies and non-profit organizations. Diagnosis of AD patients was based on NINCDS/ARDRA criteria [13]. Exclusion criteria for the ADNI data were the presence of any significant neurological disease other than AD, history of head trauma followed by persistent neurological deficits or structural brain abnormalities, psychotic features, agitation or behavioral problems within the last three months or history of alcohol or substance abuse. For most subjects, multiple follow-up MRI scans were available. For each subject only the first MRI scan was used for further analysis. The study was conducted according to the Declaration of Helsinki. Written informed consent was obtained from all participants before protocol-specific procedures were performed.

**Table 1:** Subject group characteristics for SVM.

	Controls	AD	t-test(df,t,p)
Number	79	80	-
Male/Female	41/38	40/40	-
Age (years±SD)	75.8±4.9	75.7.1±7.0	157,0.1,0.89
MMSE (score±SD)	28.7±1.7	23.6±2.2	157,16.6,<0.001
CDR (score±SD)	0.04±0.13	0.81±0.24	157,616.4,<0.001

AD Alzheimer's disease, CDR Clinical Dementia Rating, MMSE Mini Mental State Examination, SD standard deviation.

### Image preprocessing and data analysis

All image-processing steps described below were carried out using the SPM5 software package (Statistical Parametric Mapping software: http://www.fil.ion.ucl.ac.uk/spm/) implemented in Matlab 7.7 (MathWorks Inc., Sherborn, MA). SVM classification was conducted with the LIBSVM software [14] using the Matlab interface.

### MRI data

The MRI dataset included standard T1-weighted images obtained with different scanner types using the volumetric MPRAGE sequence varying in TR and TE with an in-plane resolution of 1.25×1.25 mm and 1.2 mm sagittal slice thickness. Only images obtained using 1.5T scanners were used in this study. All images were preprocessed as described on the ADNI website (http://www.loni.ucla.edu/ADNI/Data/ADNI_Data.shtml), including distortion correction and B1 non-uniformity correction.

### Preprocessing

MRI data were interpolated to an isotropic resolution of 1×1×1 mm^3^, bias-corrected for inhomogeneity artifacts, segmented and spatially normalized to an averaged size template created from all subjects using the DARTEL (Diffeomorphic Anatomical Registration Through Exponentiated Lie algebra) approach [15]. Within the normalization procedure, the data were modulated to preserve the total amount of signal in the images. The data were smoothed using a Gaussian kernel of 12 mm FWHM. This high kernel was chosen because it has been shown in previous studies investigating AD with VBM that large amount of smoothing of MRI data results in an accurate statistical evaluation of GM atrophy [16],[17]. The obtained GM images were masked twice: firstly to avoid contamination by misclassified voxels, and secondly, after the smoothing to avoid big edge effects. The mask was obtained after extensive testing by excluding all voxels in the first and the last template created by the DARTEL approach with a probability of below 0.2 for belonging to GM and including only voxels that exceed this threshold in both templates. Subsequently, all GM images were corrected for age effects using the linear regression approach described above. The statistical analysis using SVM and VBM was performed twice, with and without correction for age effects.

### SVM

Multivariate pattern classification, as described in [18], was performed with a linear kernel by identifying a separating hyperplane that maximizes the distance between different clinical groups based on whole-brain information. The optimization and cross-validation of the trained SVM was performed by using the the split half method. This procedure splits the group into two independent samples and trains the model on one of the samples for subsequent class assignation of the sample that was not included in the training procedure. Both samples are used once as training sample and once as the sample that has to be classified. This validation method enables the generalization of the trained SVM to data that have never been presented to the SVM algorithms previously. SVM classification was performed for the whole group (Table 1) twice; once with and once without age correction applied prior to SVM. The reported accuracy is the percentage of subjects correctly assigned to the clinical diagnosis in both samples. As it was expected that younger AD patients and older control subjects tend to be misclassified using SVM, AD patients and control subjects, which were misclassified with and without age correction, were compared to each other in their mean age using an independent samples t-test with a significance threshold of p<0.05 (one-tailed). Additionally, to enable an accurate evaluation of potential statistical differences in differentiation accuracies when using age-corrected compared to uncorrected data the split half procedure was repeated 60 times by randomly permuting the subjects to the training and testing groups and calculating the SVM classification accuracies for both, age-corrected and uncorrected data. The obtained distributions of accuracy values were compared to each other using an independent samples t-test with a significance threshold of p<0.05.

### VBM

To evaluate the effect of suggested age correction onto VBM analyses, differences between groups of AD patients (Table 2) and the group of healthy control subjects were assessed using voxel-wise independent samples t-tests. Thereby, pair-wise group comparisons with the control group were performed using only 25 youngest AD patients, 25 oldest AD patients or 25 AD patients fitting the mean of the control group. All three AD groups were subgroups of the AD group used for SVM comparison. For all comparisons the t-test was calculated three times: with age included as covariate, without age as covariate and using age-corrected GM images. Sex was included as covariate in all t-tests. Further, we wanted to evaluate if the proposed method for age correction leads to an over- or underestimate of atrophy in one of the AD groups. For this purpose, the control group used for SVM was split into three groups of 25 subjects each. The three groups of control subjects (Table 2) were matched for age to the three groups of AD patients. Subsequently, three VBM analyses using age-uncorrected GM images were performed comparing each of the AD groups to the age matched sample of control subjects. For these comparisons, age was additionally included as covariate. Atrophic regions were investigated with a threshold of p<0.001 (uncorrected) at the voxel level and p<0.05 (FWE corrected for multiple comparison) at the cluster level. The first threshold detects all voxels in the brain exceeding the probability of 0.001 for being significantly different between both groups. The second threshold removes all clusters smaller than a cluster size expected by chance (accounted for the number of comparisons) which additionally decreases the amount of false positive errors. Additionally, to evaluate age-related changes in healthy control subjects, voxel-wise correlations between GM densities and age were calculated using only control subjects, with sex included as covariate.

**Table 2:** Subject group characteristics for VBM.

	Young Controls	Mean Controls	Old Controls	Young AD	Mean AD	Old AD	ANOVA (df,F,p)
Number	25	25	25	25	25	25	-
Male/Female	15/10	12/13	9/16	12/13	12/13	14/11	-
Age (years±SD)	70.7±3.1	75.7±1.5	81.1±2.6	69.2±3.7	75.6±1.3	82.2±3.0	5,87.8,<0.001
MMSE (score±SD)	28.4±2.4	28.6±1.2	28.9±1.1	23.8±2.1*	23.8±2.1*	23.5±2.3*	5,48.3,<0.001
CDR (score±SD)	0.04±0.1	0.04±0.1	0.04±0.1	0.76±0.3*	0.8±0.3*	0.86±0.2*	5,112.0,<0.001

*significant difference compared to the group of age-matched control subjects. AD Alzheimer's disease, ANOVA analysis of variance, CDR Clinical Dementia Rating, MMSE Mini Mental State Examination, SD standard deviation.

Further, as the model obtained using the age correction approach described above does not fulfill the criteria for a strict cross-validation because the model estimated on control subjects is applied to the same data which were used to train the model we repeated the VBM and SVM analyses using a stricter cross-validation. Thereby, the control group was split into two equally sized subgroups. The age effect was estimated in both subgroups independently. The obtained betas for age from subgroup 1 were applied to data of subgroup 2 and vice versa. Additionally, to avoid a now possible confound in the AD group as in the control group two models have now been applied to detrend for age while in the AD only one model was used, we also split the AD group randomly into two equally sized subgroups. A model which has been estimated from only one of the control subgroups was applied on subgroup 1 from AD. Correspondingly, for the 2^nd^ AD subgroup the second model from the other control subgroup was used. This proceeding insures that the model used to detrend the data for age is only applied on data which have not been seen by the model before. For the VBM comparisons using this strict cross-validation an additional covariate was added containing the information if model 1 or 2 was used for the regression.

### Statistical analysis

Group comparisons for age and severity of dementia as measured by the MMSE (Mini Mental State Examination, [19]) and CDR (Clinical Dementia Rating Scale, [20]) between groups used for SVM were performed using independent samples t-tests (two-tailed). For the four groups used for VBM comparison on age-corrected data (all control subjects, young AD, mean AD and old AD), age, MMSE and CDR were compared by conducting ANOVAs (analyses of variance). If an ANOVA revealed a significant between-group effect, a Bonferroni t-test was calculated with a significance threshold of p<0.05 (Bonferroni corrected for multiple comparisons, two-tailed). The six groups used for pairwise age-matched VBM comparisons (young controls vs. young AD, mean controls vs. mean AD, old controls vs. old AD) were compared to each other in age, MMSE and CDR using independent samples t-tests.

Group differences for both, VBM and SVM groups, regarding sex were evaluated using a chi-square test for independent samples. The statistical analysis was performed using the commercial software package SPSS 17.0 (http://www.spss.com/statistics/).

## Results

### Clinical characteristics

The chi-square test for independent samples did not reveal a statistical differences in sex between groups used for SVM [χ^2^(1) = 0.06;p = 0.811]. MMSE scores and CDR scores differed significantly between AD patients and control subjects used for SVM (Table 1). AD patients and control subjects used for SVM did not differ in age.

There was no significant difference in sex between the three age groups of AD patients and the group consisting of all control subjects [χ^2^(3) = 2.07;p = 0.56] or between the three age groups of AD patients and the three age-matched groups of control subjects (Table 2) [χ^2^(5) = 3.44;p = 0.63] used for VBM comparisons. The ANOVA revealed significant differences in MMSE [F(150) = 85.99;p<0.001] and CDR [F(150) = 200.32;p<0.001] scores between the three AD groups and the group consisting of all control subjects used for VBM. The post-hoc tests revealed no differences in the mean MMSE and CDR scores between the three groups of dementia patients, indicating a similar severity of dementia syndrome [young AD vs. mean AD: t(48) = 0.0;p = 1.0; young AD vs. old AD: t(48) = 1.0;p = 1.0; mean AD vs. old AD: t(48) = 1.0;p = 1.0]. All three groups of AD patients had significantly lower MMSE scores compared to the control group [young AD vs. control group: t(87) = -8.1;p<0.001; mean AD vs. control group: t(87) = −8.1;p<0.001; old AD vs. control group: t(87) = −9.8;p<0.001]. As expected, the ANOVA revealed a significant group difference in age between groups used for VBM comparisons. In post-hoc tests, all three groups of AD patients differed significantly from each other [young AD vs. mean AD: t(18) =  −8.1;p<0.001; young AD vs. old AD: t(18) =  −15.0; p<0.001; mean AD vs. old AD: t(18) =  −26.6; p<0.001]. Young AD patients [t(87) =  −7.6; p<0.001] and old AD patients [t(87) =  −9.8; p<0.001] differed significantly from the control group. There was no significant difference in age between mean AD patients and control subjects [t(87) =  −0.1; p = 1.0].

For the comparison of age-matched control subjects and AD patients (young controls vs. young AD, mean controls vs. mean AD, old controls vs. old AD) independent samples t-tests did not reveal any significant differences in age [young controls vs. young AD: t(48) = 1.55;p = 0.13, mean controls vs. mean AD: t(48) = 0.0,p = 1.0, old controls vs. old AD: t(48) =  −1.36;p = 0.18]. As expected MMSE [young controls vs. young AD: t(48) = 7.22;p<0.001, mean controls vs. mean AD: t(48) = 9.86,p<0.001, old controls vs. old AD: t(48) = 10.48;p<0.001] and CDR [young controls vs. young AD: t(48) = 12.41;p<0.001, mean controls vs. mean AD: t(48) = 13.3,p<0.001, old controls vs. old AD: t(48) = 15.32;p<0.001] scores differed significantly for all comparisons of the age-matched groups.

### SVM results

**Figure 1:**
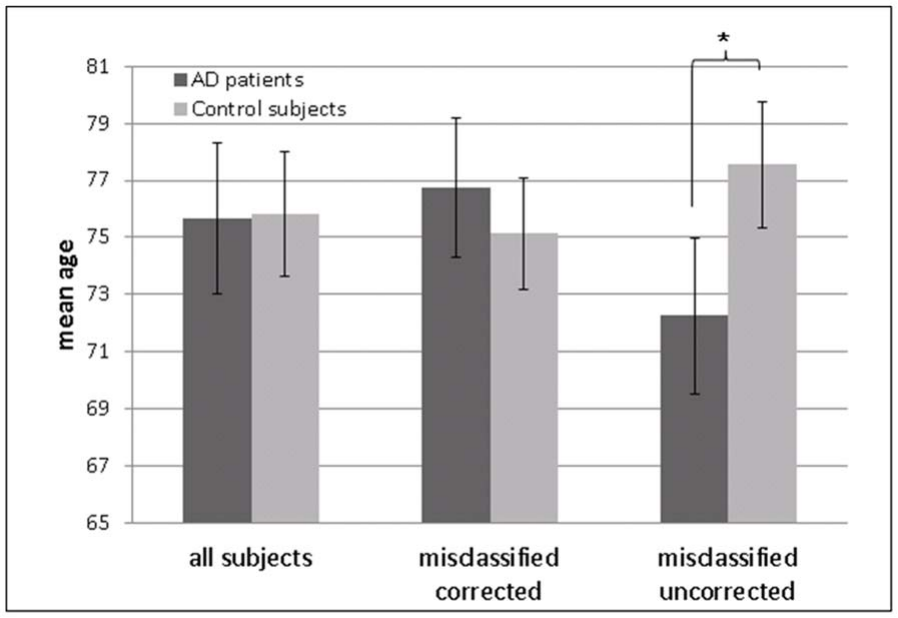
Age characteristics of missclassified subjects using SVM. Mean age of misclassified subjects in AD and control group using SVM classification with (middle two bars) and without (right two bars) age correction priorly applied. Left two bars represent the mean of all subjects in each group used for SVM. Error bars represent the standard errors of mean. AD Alzheimer's disease, SVM support vector machine classification, * significant difference between conditions.

Applying SVM to uncorrected data resulted in a high overall classification accuracy of 83.0%, which was calculated using the split half procedure. However, as expected, there was a significant difference in age between misclassified control subjects and AD patients (Figure 1). Misclassified AD patients were significantly younger than misclassified control subjects [t(25) = 2.1;p = 0.02] indicating that age has a substantial effect onto classification outcome. When SVM was applied to age-corrected data, the overall classification accuracy further increased to 85.0%. Additionally, there was no further difference in mean age between misclassified AD patients and control subjects [t(22) =  −0.69;p = 0.75].

**Figure 2:**
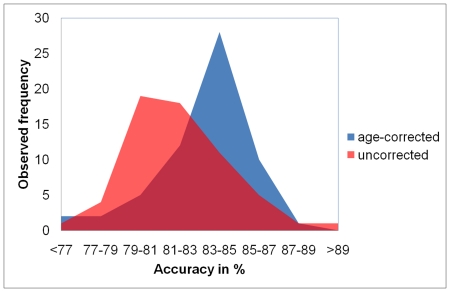
Results of permutation statistics using SVM. Results of permutation statistics using SVM split half cross-validation on uncorrected (red) and age-corrected data (blue). Observed frequency is the cumulative number of accuracies observed for a specific accuracy range.

Permutation statistic using the split half method revealed a mean classification accuracy of 81.9% using uncorrected data. Applying age-correction prior to SVM resulted in a significantly improved mean classification accuracy of 83.2% [t(118) = 2.7; p = 0.004] (Figure 2). The split half accuracy using the data obtained after the strict cross-validation (by applying an age correction model on data which have not been used to train the model) revealed a similar improvement from 82.4% using uncorrected data to 84.3% after age-correction for the same split half groups.

### VBM results

**Figure 3:**
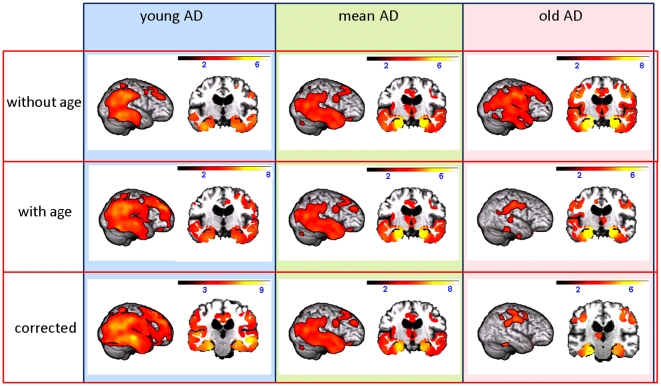
Visualization of VBM results for different age groups. GM atrophy projected onto an averaged brain detected with VBM in three groups of AD patients (columns) differing in age compared to the same group of healthy control subjects without age as covariate (upper row), with age as covariate (middle row) and after the proposed age correction (lower row). Color bars indicate the t-values. Images are thresholded with p = 0.001 on voxel level (uncorrected) and p = 0.05 on cluster level (FWE corrected). AD Alzheimer's disease, VBM voxel-based morphometry. Anatomical convention. Radiological convention.

**Figure 4:**
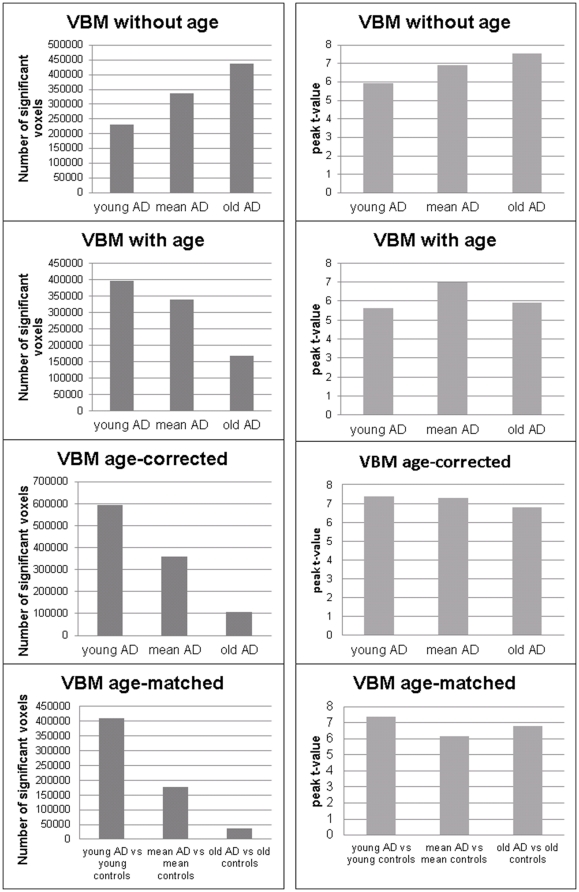
VBM results for different age groups. VBM results for the comparison of three groups of AD patients differing in age compared to the same group of control subjects without age as covariate (upper two diagrams), with age as covariate (middle two diagrams) and after the proposed age correction (lower two diagrams). Diagrams on the left represent the number of voxels detected with VBM in the three groups of AD patients. Diagrams on the right represent the peak t-values of clusters exceeding the threshold (p = 0.001 uncorrected on voxel level and p = 0.05 FWE corrected on cluster level) detected in each group of AD patients. AD Alzheimer's disease, FWE family-wise error, VBM voxel-based morphometry.

The comparison of the three groups of AD patients differing in age with the same group of control subjects without including age as covariate resulted in a differential qualitative and quantitative pattern of GM atrophy in all three groups (Figure 3 and 4). There were no voxels in the young AD group that exceeded the significance threshold. In the mean AD group, differences relative to control subjects were detected in the right hippocampus and in the right middle and inferior temporal lobe. Old AD patients showed bilaterally an extensive frontotemporal, cingulate, hippocampal and thalamic atrophy pattern compared to control subjects. Including age as covariate into VBM analyses resulted in a substantially different atrophy pattern. Young AD patients showed a bilateral atrophy in the hippocampus and middle and inferior temporal lobe. For mean AD patients, the atrophy pattern detected was very similar to the pattern detected without including age as covariate, with GM atrophy in the right hippocampus and in the right middle and inferior temporal lobe. However, there were no significant changes detected for old AD patients when age was included as covariate.

Applying age correction prior to statistical evaluation using VBM resulted in the detection of substantial differences between young AD patients and healthy control subjects, in particular parietotemporal, frontal, cuneal and hippocampal GM atrophy. A comparison of mean AD patients with control subjects after age correction resulted in a highly similar pattern of atrophy compared to VBM using uncorrected data. For old AD patients, GM atrophy after age correction were observed in the right hippocampus and in the right polar region of the temporal lobe. The comparison of the three different age groups of AD patients with age-matched control subjects revealed a similar pattern of atrophy extension to that detected using age-corrected data with younger AD patients showing the strongest GM atrophy and older AD having substantially less pronounced atrophy compared to age-matched control subjects. In these older groups a small gender imbalance might have partially biased the statistical results in this group comparison.

VBM results using the data obtained after the strict cross-validation models for age correction did not differ substantially from the results obtained using a single model to detrend for age.

**Figure 5:**
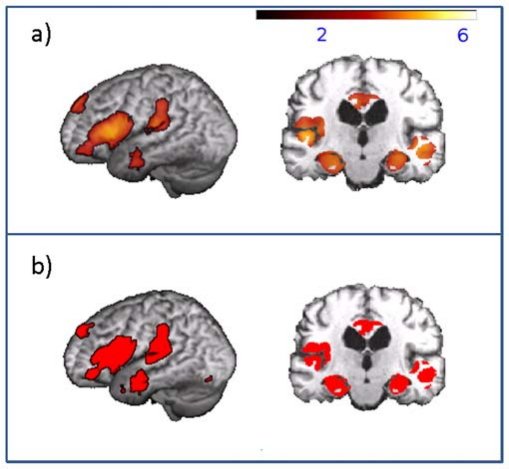
Effect of healthy aging on VBM results in AD. a) Regions showing an age-related GM atrophy in healthy control subjects detected in a correlational analysis using VBM (p = 0.001 uncorrected on voxel level and p = 0.05 FWE corrected on cluster level) plotted onto an averaged brain. Color bar represents the t-values for the correlation. b) Overlap of regions (in red) showing GM atrophy in old AD patients compared to the control group, without age included as covariate, and regions detected in the correlational analysis showing an age-related GM decline in the control group. Results are plotted onto an averaged brain. Only regions which exceeded the significance threshold (p = 0.001 uncorrected on voxel level and p = 0.05 FWE corrected on cluster level) in both analyses are shown. AD Alzheimer's disease, FWE family-wise error, GM grey matter, VBM voxel-based morphometry. Radiological convention.

Voxel-wise correlations between age and GM atrophy in healthy control subjects revealed an age-related decrease (Figure 5a) in bilateral cingulate, temporal and hippocampal regions. A further age-related decrease was observed in the right prefrontal cortex.

## Discussion

When comparing imaging data of groups of patients with healthy control subjects to investigate disease-related changes, control subjects are usually selected to match the patient groups in possible confounding variables that are expected to have an impact onto imaging data. In the further statistical evaluation, possible confounding variables are then additionally included as covariates. This straightforward procedure, although sufficient to exclude major effects of possible confounding variables, is not always applicable in clinical studies for several reasons.

In this study, we propose a methodical approach to control for effects of confounding variables like age in imaging data prior to univariate or multivariate statistical evaluation by calculating linear regression models. This is approach is similar to a method applied in some earlier studies [21],[22]. In these studies a linear regression model was applied on regional volumes and on total brain volume to control for the effect of intracranial volume prior to statistical evaluation. Thereby, age-related effects on MRI data are estimated voxel-wise using only healthy control subjects to calculate the regression coefficient. In the second step, the amount of GM atrophy explained by the age factor is removed from all data on a single subject level. To investigate the effect of the proposed age-correction method, we compared VBM and SVM results for differentiation of AD patients and healthy control subjects, with and without age correction applied prior to statistical evaluation.

SVM classification using GM values without correction resulted in very high accuracy for differentiation of AD patients and healthy control subjects, consistent with results of previous studies applying this method [18]. However, applying SVM without prior age correction resulted in a significant misclassification of younger AD patients and older control subjects indicating that age has a major impact onto differentiation accuracy using SVM. Applying age correction before SVM further increased the classification accuracy. In addtion, the two groups of misclassified patients and control subjects did not further show a difference in mean age. Although an increase of only about 2% might appear not to be noteworthy, when dealing with already very high accuracies it is more important to decrease the percentage of misclassified subjects which is in our case 17% (100%–83%: e.g. when already obtaining accuracies of 98% an additional improvement of only 1% to 99% would mean that the amount of misclassified subjects would decrease by 50% which is clinically important despite the fact that the accuracy is increased only by 1%). This error rate decreased to 15% after applying age correction which means a decrease by 12% (taking the initial 17% as baseline) in the amount of misclassified subjects which makes the improvement highly relevant for clinical application.

The improved classification accuracy after age correction and the absence of age differences between misclassified AD patients and control subjects after age correction indicate that some subjects were misclassified due to their large deviation from the mean age of the corresponding group. In younger AD patients, smaller age-related changes might have covered the disease-related effect while in older healthy control subjects, the normal age-related GM atrophy might have been misrecognized as a disease-specific alteration.

The results of VBM comparisons complement and provide further support for this interpretation. Here, three groups of AD patients differing in age were compared to the same group of control subjects. Generally, results confirmed previously reported regional atrophy patterns for AD patients [23]. When age was not included as covariate, old AD subjects showed an extensive atrophy in frontotemporal, cingulate, thalamic and hippocampal regions. In contrast, young AD patients showed substantially less extended disease-specific reductions. However, when age was correlated with GM changes in healthy control subjects, similar regions to those detected in old AD patients showed an age-related GM atrophy. Calculating an overlay of atrophy regions detected in old AD patients without including age as covariate and regions showing a normal age related decline resulted in a large overlap of changes detected in both analyses (Figure 5b). This result indicates that as expected, changes detected in old AD patients whithout including age as a covariate substantially overestimate the real amount of atrophy in this patient group. The opposite effect was observed when age was included as covariate – less disease-related GM atrophy was detected in old AD patients. Young AD patients now showed a strong decrease in GM densities in hippocampal and inferior and middle temporal regions. The amount of GM atrophy was comparable in young and mean AD patiens.

Applying the proposed age correction method prior to VBM analyses substantially improved the detection of GM atrophy in young AD patients. This group showed the most extensive age-corrected GM atrophy compared to mean AD and old AD patients. VBM performed after age correction also detected GM atrophy in old AD patients in hippocampal, inferior temporal, parietal and frontal regions although these were less extensive than in the two other groups of AD patients. For the mean AD group, GM atrophy detected without age as covariate, with age as covariate and with age correction prior to statistical evaluation did not show any substantial qualitative or quantitative differences. Additionally, the quantative and qualitative pattern detected using age-corrected data was highly similar to differences detected in comparisons of age-matched AD patients and control subjects. This similarity indicates that the application of the proposed age correction method provides a sufficient control for the effect of possible covariates like age and therefore enables a direct comparison of clinical groups with substantial initial differences in potential confounding variables. The higher cluster extension for all comparisons using age-corrected data compared to age-matched comparisons are rather a statistical artifact resulting due to different group sizes used for these comparisons. Further, the results obtained using age-corrected data are in line with previous studies indicating that less severe disease-related pathology is required with increased age to induce a similar decline in cognitive performance [11],[12]. Furthermore, as all three groups of AD patients showed a similar stage of cognitive impairment, the amounts of atrophy detected in all three groups in our study suggest a negative linear relationship between age and GM atrophy which are sufficient to induce similar cognitive impairment.

However, some very important aspects have to be considered prior to application of the proposed method to control for possible confounding variables in VBM or SVM studies. One of these major points is the group size used for the comparisons. On the one hand, the group size of the control group used for the calculation of the regression coefficients shoud be sufficiently large to provide a robust estimate of age-related changes in the total population. On the other hand, the application of the pre-regression for age does change the degrees-of-freedom in the final statistical model for VBM studies which might lead to differences in results when using smaller sample sizes. Therefore, studies using lower sample sizes should take care or account for these altered degrees-of-freedom when using the proposed method.

A further important point which has to be considered prior to application of the proposed method is the potential mutual correlation between the variable used for pre-regression and other covariates used for subsequent analysis. In our study we only used sex as an additional covariate in the subsequent VBM analysis. Furthermore, it has been shown by Good et al. [1] that the interaction between age and sex does not reach significance even in a substantially larger cohort. Therefore, we ignored this potential mutual effect in our study. Nonetheless, when applying the proposed method using any other covariates in the subsequent analysis the potential effect between these covariates and age (or any other variable for pre-regression) has to be carefully investigated. A possible option to account for mutual correlation between the covariates would be that a more complex pre-regression model is used including more than one covariate in a multiple linear regression model. However, this option has first to be carefully investigated.

Finally, the proposed method is not meant to replace classical VBM analyses which simply include possible covariates into the design matrix. The proposed option is rather meant for the studies when matching is not possible for any reason, as might be the case for example when comparing patient groups differing in more than one factor. Another option for the application of the proposed method would be a pre-regression for possible confounding effects prior to application of SVM classification.

### Conclusion and perspectives

In our study, we suggest an easily applicable approach providing the possibility to compare groups of subjects differing in specific confounding variables or to control for the effect of confounding variables in different imaging modalities in a separate step before multivariate pattern classification algorithms are applied. Using age as an example of a confounding variable in comparisons of patients with AD and healthy control subjects, we showed that applying the proposed method improves the between-group classification using SVM and the detection of univariate differences using MRI data in groups of AD patients of differing age. However, the proposed approach is not limited to age or to between-group evaluation. It can be easily applied at a group or single subject level to remove effects of any other confounding variables which might affect the statistical evaluation. However, the proposed method is not meant to replace the usual statistical approach of including possible confounding variables directly into the statistical analyses of VBM studies. If matching is easily possible which is usually the case in studies investigating healthy volunteers common statistical methods should be prefered.
